# Percutaneous Tibial Nerve Stimulation’s Impact on Sexual Function in Female Patients with Neurogenic Detrusor Overactivity, Sexual Dysfunction, and Multiple Sclerosis

**DOI:** 10.3390/jcm13206042

**Published:** 2024-10-10

**Authors:** Athanasios Zachariou, Ioannis Giannakis, Aris Kaltsas, Athanasios Zikopoulos, Charikleia Skentou, Sofoklis Stavros, Anastasios Potiris, Dimitrios Zachariou, Dimitrios Baltogiannis, Cam Hoang Nguyen Phuc, Bou Sopheap, Dung Mai Ba Tien, Nikolaos Sofikitis

**Affiliations:** 1 Department of Urology, Medical School of Ioannina, University General Hospital, 455 00 Ioannina, Greece; johgiann@hotmail.com (I.G.); dbaltog@outlook.com (D.B.); 2 Outpatient Urology Department, Physical Medicine and Rehabilitation Medicine Centre, 382 22 Volos, Greece; dimitriszaxariou@yahoo.com; 3 Third Department of Urology, Attikon University Hospital, School of Medicine, National and Kapodistrian University of Athens, 157 72 Athens, Greece; ares-kaltsas@hotmail.com; 4 Department of Obstetrics and Gynecology, Royal Cornwall Hospital, Truro TR1 3LJ, UK; athanasios.zikopoulos1@nhs.net; 5 Department of Obstetrics and Gynecology, Medical School of Ioannina, University General Hospital, 455 00 Ioannina, Greece; haraskentou@gmail.com; 6 Third Department of Obstetrics and Gynecology, University General Hospital ATTIKON, Medical School, National and Kapodistrian University of Athens, 157 72 Athens, Greece; sfstavrou@med.uoa.gr (S.S.); apotiris@med.uoa.gr (A.P.); 7 Department of Urology, Binh Dan Hospital, Ho Chi Minh City 700000, Vietnam; npchoang@gmail.com; 8 Department of Urology, Cambodia-China Friendship Preah Kossamak Hospital, Phnom Penh 120406, Cambodia; bousopheap111@gmail.com; 9 Department of Andrology, Binh Dan Hospital, Ho Chi Minh City 700000, Vietnam; maibatiendung@yahoo.com

**Keywords:** multiple sclerosis, MS, neurogenic detrusor overactivity, NDO, overactive bladder, OAB, female sexual dysfunction, FSD, posterior tibial nerve stimulation, PTNS, sexual quality of life

## Abstract

**Background/Objectives:** Multiple sclerosis (MS) frequently results in both urinary and sexual dysfunction, which significantly impairs quality of life. Conventional treatments for bladder dysfunction often prove insufficient, leading to the exploration of alternative therapies such as percutaneous tibial nerve stimulation (PTNS). This study aimed to assess the impact of PTNS on sexual function and bladder symptoms in female MS patients with neurogenic detrusor overactivity (NDO) and female sexual dysfunction (FSD). **Methods:** A total of 65 female MS patients with NDO were evaluated and underwent 12 weeks of standardized PTNS treatment. Sexual function was assessed using the Female Sexual Function Index (FSFI) and the Female Sexual Distress Scale-Revised (FSDS-R), while bladder symptoms were evaluated using the OAB-v8 questionnaire. Participants were grouped based on the presence of sexual dysfunction and distress and compared to a control group of 20 patients who declined PTNS. **Results:** Significant improvements were observed in FSFI scores across multiple domains (desire, arousal, lubrication, orgasm, satisfaction, and pain) in the treatment groups (*p* < 0.05). Additionally, 58.46% of patients showed positive responses to PTNS regarding overactive bladder symptoms (OAB-v8 score), while the control group showed no significant changes. **Conclusions:** PTNS appears to be an effective therapeutic option for improving sexual function and urinary symptoms in female MS patients with NDO and FSD, offering a promising non-invasive alternative for managing these conditions.

## 1. Introduction

Multiple sclerosis (MS) represents an autoimmune inflammatory demyelinating disease, recognized as the leading contributor to chronic neurological dysfunction [1]. It is estimated that there are approximately 2.5 million individuals worldwide affected by MS, with the majority falling within the 18–50 age group. The incidence of MS is noted to be higher in females compared to males [2]. In the clinical classification of MS, the prevailing form is the relapsing-remitting type. Some patients may transition to a secondary progressive type, while roughly 15% experience continuous progressive disability from the onset, representing the primary progressive type. Numerous genes that slightly increase disease susceptibility and several identified environmental factors combine to form MS [3]. MS manifests with a broad spectrum of symptoms, including urinary and sexual dysfunction (SD), which can profoundly affect the quality of life (QoL) of the patients [1].

Female sexual dysfunction (FSD) is a prevalent and distressing issue among women with MS, posing significant challenges to their overall well-being. Results from systematic reviews and meta-analyses show that the prevalence of sexual dysfunction in women with MS is 40–80% and the odds of developing SD in comparison with controls is 3.05 [4]. The underlying causes of FSD in MS remain subject to ongoing debate. Primary sexual dysfunction in MS is primarily caused by neurological changes due to demyelinating lesions, which impact female sexual function by decreasing genital sensation, sexual desire, and the ability to achieve orgasm. Secondary SD arises from MS-related physical symptoms such as spasticity, pain, fatigue, and bladder or bowel dysfunction, all of which indirectly interfere with sexual response [5]. Fatigue, a frequent symptom of MS, exacerbates SD in both men and women, while bladder issues can serve as indicators of neurological impairment and SD. Neuropathic pain, experienced by 60–70% of MS patients, along with physical limitations, further complicates sexual activity, with limited mobility and spasticity being major contributors [6].

Tertiary SD is influenced by psychosocial factors like mood disorders and cognitive challenges, which reduce sexual satisfaction and strain relationships. Depression, present in 27–54% of MS patients, along with anxiety and fatigue, is strongly associated with SD [7]. Research indicates that worsening depression and fatigue increase the likelihood of SD worsening. Cognitive impairments, which are common in MS, also contribute to SD, as patients with cognitive challenges report higher levels of dysfunction and reduced social interaction. Socioeconomic factors, such as employment, financial status, and education level, also correlate with SD in MS patients [7]. Sexual dysfunction may manifest at various stages of MS, often beginning early in the disease progression and becoming more prevalent as the condition advances [8].

The most prevalent urinary problem in MS patients is neurogenic detrusor overactivity (NDO), which affects up to 60% of individuals and is characterized by symptoms such as urgency and urge incontinence. Additionally, detrusor sphincter dyssynergia is observed in approximately 35% of cases, while around 25% of patients experience detrusor underactivity. NDO is a prevalent and impactful symptom complex in individuals with MS, significantly affecting their QoL. Lesions in the brain and spinal cord disrupt the bladder’s intricate neural control, leading to nerve signaling dysfunctions in the detrusor muscle and urinary sphincter. If left untreated, bladder dysfunction can lead to complications such as lower urinary tract infections, kidney damage, emotional distress, sleep problems, social isolation, and reduced quality of life [9].

Conventional treatments, including behavioral therapies and medications, may not always provide adequate relief. For patients unresponsive to pharmacological treatment for NDO or experiencing intolerable side effects, alternative options such as repeated intramuscular injections of botulinum toxin into the detrusor or sacral modulation may be considered. Surgical intervention may be an option if other means cannot achieve the desired outcomes. However, due to the progressive nature of neurological symptoms in MS and associated urological disorders, non-destructive treatment options are preferred to mitigate or delay the need for surgery [10].

Thus, alternative interventions like neuromodulation techniques have been explored as a potential possibility for addressing bladder dysfunction in individuals with MS. A literature review of 18 studies revealed that sacral neuromodulation (SNM) and posterior tibial nerve stimulation (PTNS) are particularly effective for patients with NDO, even those who do not respond to conventional treatments [11]. PTNS involves the use of electrical impulses to modulate neural pathways associated with bladder function and is considered a third-line treatment for refractory overactive bladder (OAB) [12]. Female patients suffering from MS often experience the challenges of both NDO and FSD. Despite this, there are limited available reports on the application of PTNS within the MS patient population. A non-invasive alternative to PTNS is transcutaneous tibial nerve stimulation (TTNS), which utilizes surface electrodes and may provide therapeutic advantages for individuals with MS suffering from NDO [13].

There is limited evidence comparing the safety and efficacy of TTNS and PTNS, particularly for the treatment of OAB. A review, which included 142 patients across four studies—comprising two randomized controlled trials, one retrospective study, and one before-and-after study—found similar outcomes for both TTNS and PTNS. Both methods demonstrated comparable results in terms of voiding frequency, urgency episodes, incontinence, and nocturia, with no significant differences between them [14]. The findings suggest that TTNS is equally effective as PTNS at managing OAB, with the additional benefit of no reported adverse events. However, the current evidence is insufficient, and more robust studies with larger sample sizes are needed to confirm these findings [15].

Additionally, research comparing low-frequency high-amplitude with high-frequency low-amplitude tibial nerve stimulation indicates that varying the stimulation parameters can influence treatment efficacy. However, a consensus on the optimal settings for maximizing therapeutic benefits in sexual dysfunction and bladder disorders remains to be established [16].

There are quite a few studies that have investigated the impact of PTNS on sexual dysfunction in female MS patients using validated questionnaires for both sexual function and concurrent distress, according to FSD definition. The primary objective of this pilot study was to evaluate the influence of PTNS on sexual dysfunction in female patients suffering from MS and dealing with the coexisting issues of NDO. For these reasons, specific questionnaires such as FSFI and FSDS-R were used. The secondary objective of the study was to evaluate the impact of PTNS on NDO.

## 2. Materials and Methods

From September 2021 to December 2023, 72 female patients more than 18 years of age, diagnosed with NDO due to remitting-relapsing MS, were recruited at the Rehabilitation and Physical Medicine Center EY PRATTEIN. Each participant underwent a comprehensive assessment at the beginning of the study, including a neurological and urological history and a physical examination. All patients showed damage in multiple areas, with varying degrees of nerve fiber damage to the central nervous system. Evaluations included urinalysis, urodynamic studies, and ultrasound scans of the kidneys and bladder to check for residual urine.

The research team excluded the women with a history of pelvic surgery, prior use of electrical stimulation for bladder, or recent use of TENS for other reasons. Furthermore, individuals with implanted devices such as pacemakers, defibrillators, spinal cord stimulators, or other nerve stimulators, were not eligible for the study. All women had a pregnancy test during the first session to confirm they were not pregnant. The local Ethics Committee of the Physical Medicine and Rehabilitation Centre EU PRATTEIN approved the study, which was conducted following the Declaration of Helsinki. All participants gave informed consent for the use of their data before study commencement.

An Expanded Disability Status Scale (EDSS) score < 4 was accepted as an initial inclusion criterion. EDSS is utilized to assess the level of disability and higher EDSS scores indicate a greater degree of incapacity [17]. A stable condition of MS was an essential principle for inclusion in the study. If participants experienced any worsening of their MS symptoms during the study, such as double or blurred vision, increased muscle weakness or fatigue, or loss of coordination, they were reassessed by a neurologist using the EDSS score. Women were excluded from the study if their EDSS score increased by more than 0.5 points following their initial assessment.

Participants were required to have been in a stable relationship and sexually active for at least a month prior to enrollment and throughout the study. Sexual function was assessed using the FSFI (Female Sexual Function Index) questionnaire, which includes 19 self-report questions. The FSFI measures sexual dysfunction across six domains: desire, arousal, lubrication, orgasm, satisfaction, and pain. A lower total score indicates greater dysfunction, with a score below 26.55 indicating FSD. The FSFI questionnaire is translated to and validated in the Greek language [18]. It was completed by participants during assessment visits and at the conclusion of the study.

The sexual function of female patients was also assessed with the Female Sexual Distress Scale-Revised (FSDS-R), a questionnaire designed and validated to measure distress related to inadequate or impaired sexual function. Studies using the FSDS-R have revealed that while 44% of women reported experiencing sexual complaints, only 12% reported feeling distress about their sexual function. These findings contribute to understanding the true impact of sexual complaints on women’s quality of life. A score of 11 on the FSDS-R is a reliable threshold to differentiate between women with and without sexual dysfunction [19].

All female patients presented NDO. The OAB-v8 is a screening tool used to assess how troubled patients are by four overactive bladder symptoms: voiding frequency, urgency, nocturia, and urge incontinence. The OAB-v8 has been translated to and validated in the Greek language [20]. Each item on the questionnaire is rated on a six-point Likert scale, where 0 means “not at all bothered” and 5 means “bothered a very great deal”. The total score ranges from 0 to 40, with higher scores indicating a greater burden and severity of symptoms [20].

**Figure 1:**
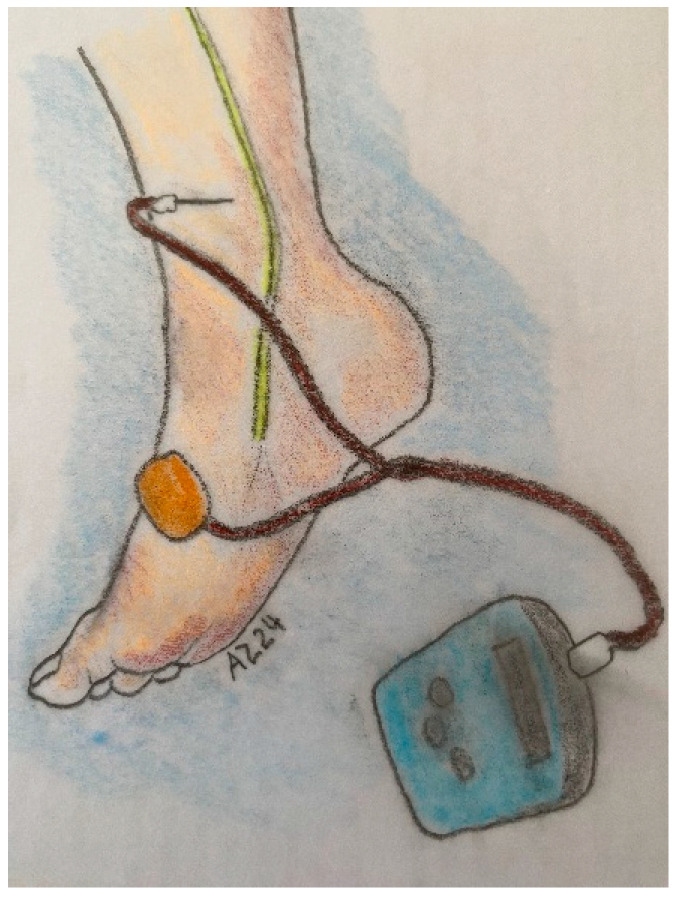
PTNS stimulation technique.

The PTNS procedure, initially introduced by McGuire et al. for treating incontinence, was later modified by Stoller and received FDA approval in 2000. The standard treatment protocol involves 12 sessions over six weeks, with each session lasting 30 min, following Stoller’s modified method. During the procedure, a needle is inserted 5 cm above and 1.5 cm behind the medial malleolus, situated between the posterior margin of the tibia and the soleus muscle. A transcutaneous adhesive electrode is placed posterior to the medial malleolus on the sole of the foot on the same side of the body (Figure 1).

Both the electrode and the needle are connected to a low-voltage (9V) electrical stimulator. The Urgent^®^ PC Stimulator (Laborie Medical Equipment, SN N-11193, Laborie Medical Technologies Corp., Portsmouth, NH, USA) was used for measurements during the neuromodulation sessions. This device is frequently cited in studies on PTNS [15,21,22,23]. The stimulation is applied at a fixed frequency of 20 Hz and a pulse width of 200 ms, with the current gradually increased from 0 to 10 mA until a motor response is observed, such as plantar flexion of the big toe or a ‘fan-shaped’ opening of the toes. This motor response is typically accompanied by a light tingling sensation in the sole of the foot [24]. A monopolar configuration was used for stimulation. The electrical current is a continuous square waveform with a duration of 200 μs and a frequency of 20 Hz. The intensity of the current is determined by the highest level tolerated by the patient [25].

The Diagnostic and Statistical Manual of Mental Disorders (DSM) defines FSD as “any sexual complaint or problem resulting from disorders of desire, arousal, orgasm, or sexual pain that causes marked distress or interpersonal difficulty”. For an issue to be classified as a dysfunction, it must cause significant distress [26].

Female patients were divided into three groups based on their FSFI total and FSDS-R score [27]. The first group, FSDa, included patients with sexual dysfunction according to the FSD definition (sexual dysfunction based on the FSFI questionnaire and sexual distress based on the FSD-R questionnaire) [27]. The second group, FSDb, comprised patients with sexual dysfunction according to the FSFI questionnaire but without sexual distress based on the FSD-R evaluation. The third group, no-FSD, consisted of patients with typical FSFI and FSDS-R total scores, characterizing a healthy female population without sexual dysfunction. It is clear that the participants were more likely categorized into the three groups (FSDa, FSDb, or no-FSD) based on their baseline FSFI and FSDS-R scores, rather than through true randomization.

Evaluations of NDO symptoms, sexual function, and disability status were conducted at both baseline and in the 12th week of the study, employing the FSFI, FSDS-R, OAB-v8, and EDSS Scale. All participants in Groups FSDa, FSDb, and no-FSD received a standardized treatment protocol consisting of two 30 min PTNS sessions per week for six weeks, followed by one session per week for an additional six weeks. A female was considered an objective responder if she had an FSFI score lower than 26.55 and an increase of more than 20% [24,28]. For OAB, objective responders were defined as females who showed a reduction of at least 10 points in the OAB-v8 score [29].

Sample size calculation can be guided by previous literature, pilot studies and past clinical experience. The target sample size for this study was initially a minimum of 20 patients, divided into two groups with ten subjects each, in line with the sample sizes used in other neuromodulation pilot studies [30,31,32,33]. Dunya et al., in a similar study, also included 10 patients in the TTNS Group [28]. Carilli et al. studied PTNS for the treatment of NDO in MS patients and included 29 patients in the study group and none in the Control Group [34]. Administrative data from the Greek nationwide medicine prescription database indicated that, with a 95% confidence level and a 5% margin of error, a sample size of 15 patients per group would be necessary [35].

In this study, we recruited 72 female MS patients; however, only 65 participants were included in the final statistical analysis due to dropout and exclusion criteria. These participants were divided into three study groups to avoid sample size limitations. The primary endpoint was the FSFI and FSDS-R score after PTNS treatment in MS with FSD and NDO, while the secondary endpoint was the change in the OAB-v8 questionnaire.

A posterior power analysis was also performed, assuming a small to medium effect size and a significance level of 0.05. The calculated power was 40.35%, suggesting that the study was underpowered for detecting smaller effect sizes. For future studies, a larger sample size would be required to reach the commonly accepted power threshold of 80%.

Data were analyzed using IBM SPSS Statistics for Windows, Version 20.0. Descriptive statistics were calculated for all variables. The Shapiro–Wilk test was used to assess the normality of data distribution. The related-samples Wilcoxon signed-rank test was chosen due to the non-normal distribution of data, as determined by the Shapiro-Wilk test. While repeated-measures ANOVA or the Friedman test could offer a more comprehensive analysis of differences across three groups and multiple time points, the decision to use a non-parametric approach was based on the skewed data distribution and small sample size.

Considering the comparison of three groups (FSDa, FSDb, and no-FSD), it is recommended that the Kruskal–Wallis test, a non-parametric alternative to ANOVA, is more suitable for such analyses. The Kruskal–Wallis test has now been employed to evaluate differences across the three groups.

Initially, no corrections for multiple comparisons, such as the Bonferroni correction, were applied due to the exploratory nature of the study and the small sample size. However, it is acknowledged that the lack of such corrections may increase the likelihood of type I errors. For future studies with larger sample sizes, applying correction methods will be necessary to minimize the risk of false positives.

Effect sizes have been calculated for each comparison to provide additional insight into the clinical significance of the findings. For non-parametric tests, the effect size was calculated using the r value, while for parametric comparisons, Cohen’s d was employed. A *p*-value of less than 0.05 was considered statistically significant. Values are expressed as mean ± standard deviation unless otherwise specified.

**Figure 2:**
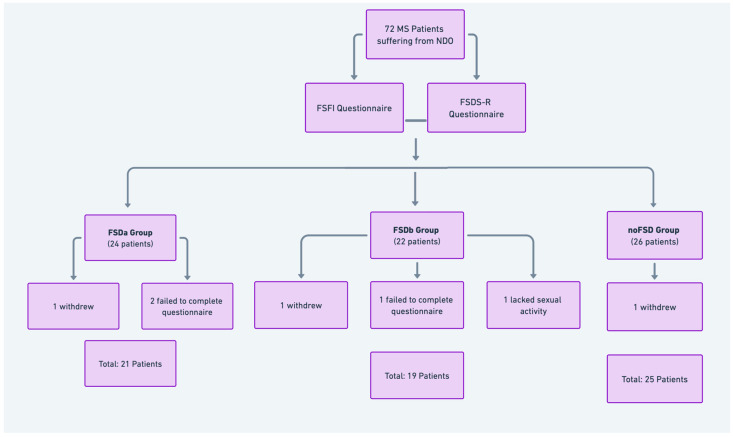
CONSORT flow diagram illustrating the selection and allocation of study participants.

Figure 2 presents a flowchart detailing the selection process of the study population, including the number of patients screened, excluded, enrolled, allocated to groups, and analyzed.

## 3. Results

Out of the 72 enrolled females, 65 were evaluated and included in the statistical analysis, divided into three groups (FSDa, FSDb, and no-FSD Group). Seven eligible participants were excluded during the follow-up period: three withdrew from the study, three failed to complete the required protocol questionnaires, and one was excluded due to a lack of sexual activity during treatment.

The average age of the patients was 42.9 ± 6.72 years. The socio-demographic characteristics were comparable between the study groups (*p* > 0.05). All participants had relapsing-remitting MS, with a disease duration of more than seven years. Bladder symptoms had been present for approximately five years. All female participants exhibited a stable MS condition, with no deterioration in their EDSS scores. Clinically, the patients in both study groups were similar (*p* > 0.05) (Table 1).

We formed three patient groups based on the FSFI total score and FSDS-R score. The FSDa group, consisting of 21 patients, represented women with sexual dysfunction according to the FSD definition. The FSDb group included 19 patients with sexual dysfunction but without sexual distress. The third group, no-FSD, consisted of 25 patients with normal FSFI and FSDS-R scores, representing a healthy female population without sexual dysfunction. The changes to the FSFI domains score are described in Table 2.

The FSDS-R Questionnaire was evaluated in the FSDa Group to identify changes before and after treatment. At the end of the PTNS protocol, 9 patients out of 21 (42.85%) had no sexual distress. The FSDS-R questionnaire yielded normal values for the other two groups, the FSDb and no-FSD groups.

Thirty-eight out of 65 individuals (58.46%) were identified as subjective responders for OAB. Significant enhancements were observed in the overall urinary function (*p* < 0.05). The changes in OAB-v8 score are described in Table 3.

The study found that only minor side effects were noted. Of the patients, two experienced aggravated urinary symptoms, but these symptoms were transient and resolved on their own later. Five patients reported temporary pain or numbness during stimulation, which was successfully managed by reducing the stimulation intensity.

## 4. Discussion

MS is a chronic neurological disorder that often results in neuro-urological issues, including NDO, detrusor sphincter dyssynergia (DSD), and hypocontractility. NDO is identified by symptoms including urgency, frequent urination, nocturia, and urge-related urinary incontinence. These symptoms have a profound impact on the QoL, with urinary dysfunctions being highly prevalent among MS patients. Furthermore, these symptoms are frequently associated with sexual dysfunction [36]. Around 80% of individuals with MS experience bladder dysfunction within a decade of their diagnosis, with many reporting these issues at the onset of the disease.

Female sexual dysfunction is a multifaceted condition encompassing issues such as decreased libido, arousal difficulties, and pain during intercourse. The overlap between OAB and FSD suggests that addressing lower urinary tract symptoms (LUTS) may concurrently benefit sexual function [37]. PTNS, by alleviating OAB symptoms, indirectly enhances sexual function by reducing the physical and psychological burden of bladder dysfunction.

PTNS has gained recognition as an effective treatment option for different types of bladder dysfunction, including NDO or OAB, female sexual dysfunction and bladder complications related to MS [38]. PTNS uses a needle electrode to stimulate the tibial nerve located near the ankle. The precise mechanisms by which PTNS improves bladder and sexual functions are not entirely understood, but several hypotheses have been proposed. PTNS likely modulates both afferent and efferent neural pathways involved in bladder control [39]. Stimulation of the tibial nerve may inhibit bladder activity by depolarizing sacral and lumbar afferent fibers, which subsequently inhibit preganglionic bladder motor neurons in the spinal cord [40]. This neural modulation could also impact the neural circuits involved in sexual arousal and response, explaining the observed improvements in sexual function.

Our study confirms the therapeutic effects of PTNS on female sexual function. The significant improvements in FSFI scores observed align with numerous studies that highlight PTNS’s potential to improve sexual dysfunction. Additionally, our study is one of the few to use the FSDS-R questionnaire, providing robust and reliable results. In line with our results, Musco et al. (2016) conducted a study that revealed significant improvements in sexual function among women with OAB following PTNS therapy. In this study, 41 women with idiopathic dry OAB underwent 12 weekly sessions of PTNS. Sexual function was evaluated using the FSFI questionnaire only, and the findings indicated considerable enhancements across all domains, including desire, arousal, lubrication, orgasm, satisfaction, and pain. Remarkably, 43% of the women who initially had FSD demonstrated measurable improvements in their FSFI scores after receiving PTNS treatment [24]. Furthermore, in a systematic review and meta-analysis, Kershaw et al. (2019) confirmed the positive effect of PTNS on sexual function, highlighting significant improvements across FSFI domains. Although the included studies were relatively small, the pooled data underscored the therapeutic potential of PTNS in managing both bladder and sexual dysfunction [41].

Additionally, Finazzi-Agro et al. (2009) found that PTNS modified brain activity in women with refractory OAB suggesting that its efficacy might involve central neural mechanisms beyond peripheral modulation [42]. This central involvement could explain the broader therapeutic benefits of PTNS, including the improvements in sexual function observed in various studies.

Carilli et al. (2023) investigated the effectiveness of PTNS in treating NDO in patients with MS who have not responded to pharmacological and behavioral therapies. The study enrolled 33 patients and found that 72.4% of those who underwent PTNS showed significant improvements in bladder function, as evidenced by reduced urgency episodes and improved questionnaire scores. The results suggest that PTNS is a promising, minimally invasive treatment for MS-related NDO [34].

Rahnama’i et al. reviewed neuromodulatory treatments for OAB in MS patients. PTNS was highlighted as an effective treatment for reducing urinary symptoms and improving QoL in this patient group. The review included studies that demonstrated PTNS’s efficacy in decreasing daytime frequency, nocturia, urgency episodes, and residual urine volume in MS patients. These improvements in bladder control likely contribute to enhanced sexual function by reducing the psychological and physical burdens associated with urinary symptoms [38].

Dunya et al. investigated the impact of two non-invasive treatment methods—transcutaneous tibial nerve stimulation (TTNS) and pelvic floor muscle training (PFMT)—on FSD in women with MS who also experienced OAB symptoms. The research included 30 female MS patients, divided into two groups: one receiving TTNS and the other PFMT with biofeedback. The results showed that both TTNS and PFMT significantly improved sexual function and OAB symptoms. Specifically, the TTNS group saw a 70% improvement in FSFI scores, while the PFMT group showed a 40% improvement. Additionally, PFMT also significantly enhanced sexual quality of life. However, when comparing the two groups, there were no significant differences between TTNS and PFMT in terms of their impact on sexual function, OAB symptoms, or sexual quality of life [43].

Zimmerman et al. evaluated the effectiveness of transcutaneous electrical nerve stimulation (TENS) applied to the dorsal genital nerve (DGNS) and PTNS as a treatment for FSD, specifically focusing on improving genital arousal deficits in women without bladder problems. The results indicated significant improvements in overall sexual function, particularly in the FSFI sub-domains of arousal, lubrication, and orgasm. The study observed that these improvements persisted across all time points, although there was a slight decline after the six-week washout period, suggesting that maintenance sessions might be necessary to sustain the benefits. Interestingly, the PTNS group showed a greater improvement in sexual functioning compared to the DGNS group, although the small sample size limited the statistical comparison [30].

Thus, our results align well with the broader body of research that confirms the efficacy of PTNS in improving both bladder and sexual dysfunctions, particularly in women with MS. These findings not only reinforce PTNS as a promising treatment for managing FSD in MS patients but also highlight its broader role in reducing the physical and psychological burdens associated with neurogenic bladder and sexual dysfunction.

The optimal PTNS session schedule for NDO is still under investigation and remains a topic of debate. Some patients may require continuous sessions to maintain the benefits. Additionally, not all patients respond to PTNS treatment, and the predictors of response are not well defined. Further studies are needed to identify the characteristics of responders and non-responders. Previous research suggests that patients with OAB who have a very low baseline cystometric capacity are more likely to be unresponsive to PTNS [6].

A limitation of the study is the absence of a follow-up period, meaning the long-term efficacy of PTNS is still under investigation. Additionally, while 65 participants were included in the final analysis, the small sample size limits the generality of the findings. The study also lacks a control group, which would have provided a more robust comparison. Furthermore, the patient groups were formed based on their baseline PSFI and FSDS-R scores, as per existing definitions of sexual dysfunction, which made randomization and blinding difficult to implement. As a result, potential biases cannot be entirely ruled out. Finally, the EDSS cut-off score used in this study may not be applicable to all MS populations, limiting the broader application of these findings.

Despite these limitations, it is important to note that PTNS has a favorable safety profile, with minimal and transient side effects such as mild pain or numbness at the needle insertion site.

## 5. Conclusions

PTNS has emerged as a valuable treatment option for improving both sexual function and urinary symptoms in female MS patients with NDO and FSD. The significant improvements observed in FSFI scores across multiple domains suggest that PTNS may address sexual dysfunction independently of urinary symptom improvement. These findings support the use of PTNS as a promising non-invasive alternative for managing these conditions, although further randomized controlled trials with larger sample sizes and longer follow-up periods are needed to confirm these results.

## Figures and Tables

**Table 1:** Sociodemographic and clinical characteristics of the female MS participants (mean ± standard deviation).

Characteristics	MS Patients (*n* = 65)
Age	43.1 ± 7.34
BMI	26.4 ± 5.15
Children	1.43 ± 0.62
Time from diagnosis	7.23 ± 4.76
EDSS	3.21 ± 0.54
Time urinary symptoms	5.52 ± 2.89

MS: Multiple sclerosis, BMI: Body mass index, EDSS: Expanded disability status scale.

**Table 2:** Results of the FSFI Questionnaire in MS patient groups before and after treatment with PTNS and Control Group.

	Desire	Arousal	Lubrication	Orgasm	Satisfaction	Pain	Total
FSDa Group, 21 p							
Before Treatment							
Median	2.31	2.79	2.87	3.29	3.01	3.48	17.75
Interquartile range	1.94–2.73	2.31–3.21	2.43–3.92	2.73–3.46	2.73–3.46	3.01–4.07	15.74–20.11
After treatment							
Median	3.34	3.74	3.99	4.01	4.03	4.24	23.35
Interquartile range	2.87–4.05	3.36–4.34	3.55–4.56	3.49–4.63	3.56–4.56	3.73–4.72	20.76–25.39
*p*	<0.05	<0.05	<0.05	<0.05	<0.05	<0.05	<0.05
FSDb Group, 19 p							
Before Treatment							
Median	2.41	2.97	2.88	3.37	3.31	3.47	18.41
Interquartile range	1.85–3.08	2.48–3.69	2.33–3.45	2.94–3.86	2.83–3.85	2.94–3.95	15.95–20.72
After treatment							
Median	3.32	3.61	4.05	3.96	4.06	4.28	23.28
Interquartile range	2.87–4.17	3.08–4.24	3.61–4.81	3.49–4.48	3.62–4.73	3.77–4.88	20.68–25.74
*p*	<0.05	<0.05	<0.05	<0.05	<0.05	<0.05	<0.05
No-FSD, 25 p							
Before Treatment							
Median	4.05	4.46	4.72	4.76	4.91	5.54	28.44
Interquartile range	3.69–4.57	4.02–5.07	4.33–5.17	4.41–5.24	4.49–5.27	5.21–5.71	27.42–31.09
After treatment							
Median	4.08	4.51	4.81	4.82	5.58	5.57	29.37
Interquartile range	3.79–4.59	4.13–5.16	4.56–5.10	4.45–5.21	5.24–5.87	5.27–5.89	27.61–31.34
*p*	>0.05	>0.05	>0.05	>0.05	<0.05	>0.05	>0.05

FSD: Female sexual dysfunction, *p*-value: probability value.

**Table 3:** The OAB-v8 score before and after the treatment in the study groups.

OAB-v8 Score	
FSDa Group, 21 p	
Mean ± SD (before treatment)	29.45 ± 4.34
Mean ± SD (after treatment)	21.22 ± 3.81
*p*	<0.05
FSDb Group, 19 p	
Mean ± SD (before treatment)	28.12 ± 3.96
Mean ± SD (after treatment)	20.65 ± 3.57
*p*	<0.05
No-FSD, 25 p	
Mean ± SD (before treatment)	28.78 ± 4.10
Mean ± SD (after treatment)	19.96 ± 3.41
*p*	<0.05

FSD: Female sexual dysfunction, OAB: overactive bladder symptoms, SD: Standard deviation, *p*-value: probability value.

## Data Availability

The original contributions presented in the study are included in the article, further inquiries can be directed to the corresponding authors.
